# Unusual MRI Findings in African *Trypanosoma brucei gambiense* Trypanosomiasis: Dentate Nuclei and Hypothalamic Lesions

**DOI:** 10.4269/ajtmh.19-0204

**Published:** 2020-01

**Authors:** Monique Boukobza, Sylvie Lariven, Sandrine Houzé, Jean-Pierre Laissy

**Affiliations:** 1 Department of Radiology, Assistance Publique-Hôpitaux de Paris, Bichat University Hospital, Paris, France;; 2 Infectious and Tropical Diseases Department, Assistance Publique-Hôpitaux de Paris, Bichat-Claude Bernard University Hospital, Paris, France;; 3 Parasitology and Mycology Laboratory, Assistance Publique-Hôpitaux de Paris, Bichat-Claude Bernard University Hospital, Paris, France;; 4 INSERM U1148, Paris, France;; 5 University Paris 7, Bichat Hospital, Paris, France

A 56-year-old male emigrated from Mali 6 years ago. He was admitted because of somnolence, confusion, decreased concentration, gait instability and weight loss over a two month period. He also spent his holidays in Mali in 2016. Examination revealed cervical and axillary lymphadenopathy and wide-based gait.

**Figure 1.**
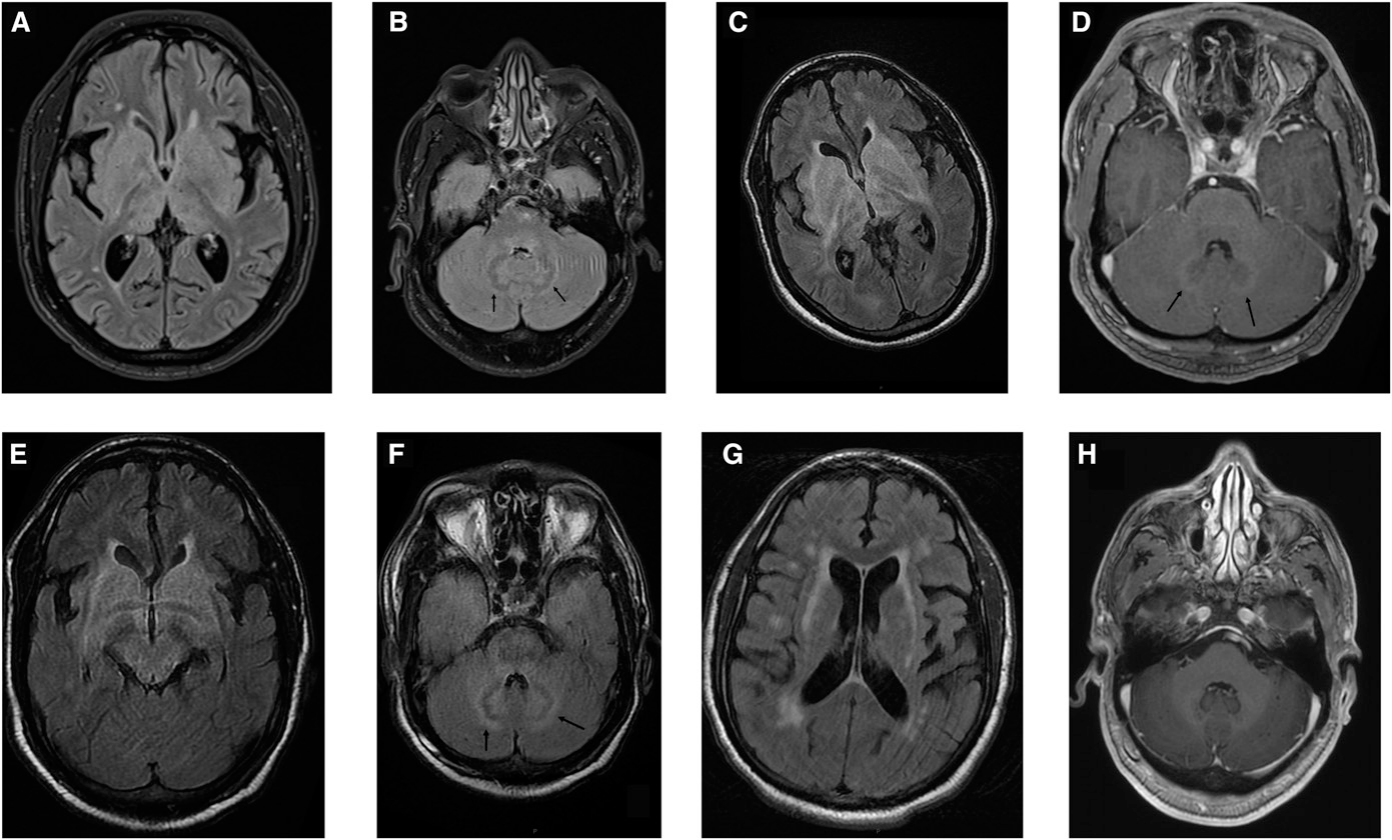
Brain MRI before and after treatment. (**A** and **B**) At admission, MRI shows on fluid-attenuated inversion recovery (FLAIR) sequence diffuse hyperintensity in both lenticular nuclei, caudate nuclei, thalami, and internal capsule (**A**) and hypointense signal of both dentate nuclei (DN) with a thin hyperintense blurring at the external limit of the DN (**B**, arrows) as well as hyperintensity at the dorsal part of the pons. (**C**–**F**) Follow-up MRI before treatment performed 7 weeks after the first MR examination demonstrates interval progression of the FLAIR hyperintensity signal in both lenticular nuclei, caudate nuclei, thalami, and internal capsule, and also of the external capsule and the hypothalamus (**C**). An hyperintensity on FLAIR sequence is also present in the ventral and dorsal pons and of the whole midbrain (**D**). Fluid-attenuated inversion recovery sequence also shows an interval increase in the hypointense signal of both DN and of the hyperintense halo (**E**, arrows) and areas of hyperintensity at the anterior and dorsal pons. Diffusion sequence was unremarkable. On post-contrast T1 sequence, there is an enhancement at the periphery of the DN (**F** arrows). (**G** and **H**) Follow-up MRI posttreatment. Follow-up MRI 1 year after initiation of treatment shows residual FLAIR hyperintensity in the external capsules. There has been new interval appearance of patchy hyperintensities of the subcortical and deep white matter, not previously observed (**G**). The enhancement of DN after contrast has disappeared (**H**).

Non-contrast brain computed tomography (CT) was normal. Cerebrospinal fluid (CSF) analysis showed WBC of 107/mm^3^ (95% lymphocytes) and protein level of 0.9 g/L. CSF bacterial, fungal, and viral cultures were all negative. Thoracic and abdominopelvic CT showed thoracic and subdiaphragmatic adenopathies. Magnetic resonace imaging (MRI) revealed hyposignal of the dentate nuclei (DN) on fluid-attenuated inversion recovery (FLAIR) sequence (Figure 1A and B). In addition, many other symmetrical areas displayed abnormal FLAIR hypersignal.

The patient’s consciousness deteriorated (Glasgow coma score [GCS] of 11/15, then GCS 8) and fever of 38.9°C. Blood and CSF polymerase chain reaction were positive for *Trypanosoma* sp. Blood smears and bone marrow aspirates showed numerous *Trypanosoma*. Brain MRI revealed a marked hypointense signal of the DN on FLAIR, surrounded by a hyperintense halo, also involving the inner cerebellar cortex*.* High FLAIR signal was seen in the corona radiata, midbrain, pons, medulla, cerebellar white matter (WM), caudate nuclei (CN), and hypothalamus, which were not observed previously in humans. Dentate nuclei exhibited a faint contrast enhancement (Figure 1C–F). Treatment by nifurtimox (oral 15 mg/kg/day every 8 hours) and eflornithine (100 mg/kg iv every 6 hours) was carried out over 7 days. Clinical and MRI follow-up at 1, 2, and 5 months posttreatment demonstrated gradual improvement. At 6 months, the clinical examination was normalized. No residual or new clinical symptom was found at one-and-half-year follow-up, and MRI examination demonstrated near-complete resolution of these lesions, except for the external capsule involvement, but new patchy FLAIR hyperintensities in the subcortical and deep WM, not previously observed (Figure 1G–H).

In patients with neurological deficits, MRI demonstrates specific findings (11 reported cases with enough MRI data obtained before melarsoprol treatment was initiated, including ours).^1–5^ Large WM hyperintensities are the most common finding (9/11; internal/external capsule: 5/11 and cerebellar WM hyperintensities: 3/11). The BG is also often concerned (lenticular nuclei [LN]: 9/11) as the thalami (6/11). More rarely the BS is involved: FLAIR hyperintensities at the central pons (4/11) (present case^2–4^), at the anterior aspect of the midbrain (3/11) (present case^1^), and at the medulla oblongata (present case) have been reported. Other rare lesions, appearing as FLAIR hyperintensities, involve the CN (3/11) (present case^1–4^), bilateral middle cerebellar peduncles (2/11),^1–5^ and the corpus callosum.^5^ Another interesting finding was the symmetric distribution of the lesions on MRI in 6/11 cases (present case^3–5^) and almost symmetric in two other cases.^1^

Different rare entities may affect the DN. Among them, metabolic, toxic and drug induced (metronidazole, cycloserine, isoniazid toxicity, and methyl bromide), inflammatory, and enterovirus 71 infection in children can lead to DN damage. Our patient had no evident intoxication and was not under previous treatment. In addition, in the present case, the MRI patterns of DN signal changes and of the associated brain lesions, along with the context, narrow down the differential diagnosis.

Our case highlights several new imaging findings of the disease, some of these findings were previously only described in postmortem autopsy studies^6^ but not on imaging: MRI involvement of the DN and hypothalamus and faint contrast enhancement of the DN (the only enhancing lesion) which appeared on follow-up imaging before treatment initiation. The timing of both appearance and regression of the DN and hypothalamic anomalies were the same as the other lesions on follow-up MRI.

Furthermore, the present case shows a correlation between the clinical and radiological findings: involvement of the cerebellum, including the DN, was correlated with clinical findings of somnolence, memory, and gait impairment.
